# Ceramide Kinase Inhibition Blocks IGF-1-Mediated Survival of Otic Neurosensory Progenitors by Impairing AKT Phosphorylation

**DOI:** 10.3389/fcell.2021.678760

**Published:** 2021-06-04

**Authors:** Yolanda León, Marta Magariños, Isabel Varela-Nieto

**Affiliations:** ^1^Instituto de Investigaciones Biomédicas “Alberto Sols”, CSIC-UAM, Madrid, Spain; ^2^Departamento de Biología, Universidad Autónoma de Madrid, Madrid, Spain; ^3^CIBERER, Unit 761, CIBER, ISCIII, Madrid, Spain

**Keywords:** ceramide metabolism enzymes, development, sphingolipids, NVP-231, otic progenitors, PF-543

## Abstract

Sphingolipids are bioactive lipid components of cell membranes with important signal transduction functions in health and disease. Ceramide is the central building block for sphingolipid biosynthesis and is processed to form structurally and functionally distinct sphingolipids. Ceramide can be phosphorylated by ceramide kinase (CERK) to generate ceramide-1-phosphate, a cytoprotective signaling molecule that has been widely studied in multiple tissues and organs, including the developing otocyst. However, little is known about ceramide kinase regulation during inner ear development. Using chicken otocysts, we show that genes for CERK and other enzymes of ceramide metabolism are expressed during the early stages of inner ear development and that *CERK* is developmentally regulated at the otic vesicle stage. To explore its role in inner ear morphogenesis, we blocked CERK activity in organotypic cultures of otic vesicles with a specific inhibitor. Inhibition of CERK activity impaired proliferation and promoted apoptosis of epithelial otic progenitors. CERK inhibition also compromised neurogenesis of the acoustic-vestibular ganglion. Insulin-like growth factor-1 (IGF-1) is a key factor for proliferation, survival and differentiation in the chicken otocyst. CERK inhibition decreased IGF-1-induced AKT phosphorylation and blocked IGF-1-induced cell survival. Overall, our data suggest that CERK is activated as a central element in the network of anti-apoptotic pro-survival pathways elicited by IGF-1 during early inner ear development.

## Introduction

The vertebrate inner ear contains the organs responsible for the perception of sound and balance whose functions are mediated by specialized mechanoreceptor cells maintained in position by support cells and innervated by the terminations of the auditory and vestibular ganglia (Magariños et al., 2012). Loss of mechanoreceptor cells or their malfunction causes severe sensory disturbances of great prevalence in the case of deafness (WHO: 1 in 4 people projected to have hearing problems by 2050, WHO, 2021). As these postmitotic sensory cells do not regenerate in mammals, but do so birds, there is great interest in understanding the molecular and genetic bases of the formation of these highly differentiated cells during development, including in experimental bird models (Shi and Edge, 2013; Roccio et al., 2019).

The inner ear originates from the otic placode, an ectodermal thickening that arises in the hindbrain and invaginates and closes to form the otic vesicle or otocyst. The otic vesicle, a transitory fluid-filled structure lined by a pseudo-stratified epithelium, is considered the primordium of the inner ear, as its epithelial cells differentiate and generate most of the cell types of the adult inner ear (Torres and Giráldez, 1998; Fekete and Wu, 2002). The transition from otic progenitors to mature inner ear cells occurs through the spatiotemporal regulation of proliferation, differentiation, migration, apoptosis, senescence, and autophagic processes, which creates the highly organized architecture of the adult sensory organ (Sánchez-Calderón et al., 2007; Varela-Nieto et al., 2019). The sensory epithelium, named the organ of Corti in mammals and the basilar papilla in birds, is connected to the brain through the VIIIth cranial nerve. The otic epithelium is also a source of neural progenitors, specifically the ventromedial region from where neural progenitors delaminate and migrate to form the neurons of the acoustic-vestibular ganglion (AVG) (D’Amico-Martel, 1982; Bell et al., 2008).

The otocyst can be isolated and cultured *ex vivo* and maintains the traits of *in vivo* otic development, being a powerful organotypic model system to study growth factor requirements and early developmental events (Represa et al., 1988). In chicken organotypic otocysts it has been demonstrated that insulin-like growth factor type 1 (IGF-1) has a key role in otic vesicle morphogenesis and growth (León et al., 1995, 1998; Frago et al., 2003), and participates in AVG neurogenesis (Camarero et al., 2003). Homozygous mutations in the human and mouse genes encoding IGF-1 cause sensorineural hearing loss (Woods et al., 1996; Cediel et al., 2006). IGF-1 also has a protective function in the mammalian cochlea by promoting the survival and maintenance of hair sensory cells and synapses (Gao et al., 2020). Accordingly, it has been used for the treatment of certain types of sensorineural hearing loss (Murillo-Cuesta et al., 2011; Yamahara et al., 2015). Engagement of IGF-1 with its high affinity receptor IGF1R activates PI3K-AKT signaling, the main downstream target in *ex vivo* chicken (Frago et al., 2003; Aburto et al., 2012) and murine (Okano et al., 2011) organotypic cultures. Compared with wild-type mice, embryonic cochlea of *Igf1* null mice show a low activation of this pathway (Sanchez-Calderon et al., 2010). The inactivation of PI3K-AKT signaling is also related to defects in the zebrafish inner ear (Xia et al., 2019). While there is a strong correlation between IGF-1 and the PI3K-AKT pathway during ear development, the role of the classical proliferative RAF-MEK-ERK pathway is less clear (Magariños et al., 2010; Okano et al., 2011). Concurrent with proliferation, developmental apoptosis is highly regulated during inner ear development and, therefore, signals counteracting the pro-survival and proliferative actions IGF-1 are also in place. Synthetic sphingolipid C2-ceramide is a potent activator of apoptosis and inhibits IGF-1-induced proliferation by blocking AKT activation (Frago et al., 1998, 2003). The pro-apoptotic actions of ceramide are regulated by its direct phosphorylation by ceramide kinase (CERK) to form the pro-survival ceramide derivative ceramide-1-phosphate (C1P) (Sugiura et al., 2002). Short-chain C1P is a cytoprotector in cultured chicken otic vesicles and it has been proposed that IGF-1 could be an activator of CERK to process intracellular ceramide to C1P (Frago et al., 1998). As a central component and precursor in sphingolipid metabolism, ceramide can also be processed to sphingosine-1-phosphate (S1P) involved in the differentiation of murine auditory progenitors (Cencetti et al., 2019). Sphingosine-kinase (*Sphk*) null mice have severe neural defects and are embryonic lethal (Mizugishi et al., 2005). By contrast, *Cerk* null mice survive to adulthood (Graf et al., 2008b). Nothing is yet known about the expression pattern of these enzymes during inner ear development and the hearing phenotype of the *Cerk* null mouse has not been reported. Interestingly, however, *Cerk* null mice present chronic inflammation (Lamour et al., 2011; Suzuki et al., 2018) and uncontrolled inflammation is one of the mechanisms underlying hearing loss (Prasad and Bondy, 2020).

We report here the expression pattern of the principal genes involved in ceramide metabolism in the early developing chicken inner ear. Our finding of *CERK* expression in otocysts prompted us to test its role as a potential mediator of otic development and IGF-1 signaling. To this end, we used the specific pharmacological CERK inhibitor NVP-231 which decreases the levels of C1P (Graf et al., 2008a). We found that inhibition of CERK activity in otic vesicle cultures decreased otic vesicle size and reduced progenitor cell proliferation, and this was accompanied by elevated cell cycle arrest and apoptosis, and compromised AVG neurogenesis, demonstrating that CERK is essential for otocyst development and AVG neurogenesis. Finally, CERK inhibition reduced AKT phosphorylation and impaired the effect of exogenously added IGF-1, strongly suggesting that CERK participates in the IGF-1 signaling network during inner ear development.

## Materials and Methods

All chemical reagents were purchased from Sigma-Aldrich (Sigma-Aldrich, Saint Louis, MO, United States) unless specified otherwise.

### Organotypic *ex vivo* Cultures

Fertilized eggs were purchased from a local farm and incubated at 38°C in a humidified atmosphere. Otic vesicles were dissected from HH18 (<3 days) chicken embryos that were staged according to Hamburger and Hamilton’s classification (HH) (Hamburger and Hamilton, 1992).

Standard organotypic culture media consisted of M199 medium with Earle’s salts, supplemented with 2 mM glutamine (Gibco, Paisley, United Kingdom), 50 IU/ml penicillin (Ern, Barcelona, Spain) and 50 μg/ml streptomycin (CEPA, Madrid, Spain). Isolation and culture of otic vesicles was performed following a published protocol (León et al., 1995). Briefly, otic vesicles were dissected free of the surrounding mesenchyme, transferred to four-well culture plates (Thermo Fisher Scientific, Roskilde, Denmark) and incubated at 37°C for 20 h in a water-saturated atmosphere with 5% CO_2_. Culture treatments included serum-free medium (SFM, control condition), 10 nM IGF-1 (recombinant IGF-I; Roche Molecular Biochemicals, Basel, Switzerland), the CERK inhibitor adamantane-1-carboxylic acid (2-benzoylamino-benzothiazol-6-yl)amide (NVP-231, hereafter called CKi) (Tocris Bioscience, Bristol, United Kingdom) prepared in DMSO, and the sphingosine kinase 1 (SPHK1) inhibitor PF-543 hydrochloride (Tocris Bioscience). Both inhibitors are effective at nanomolar concentrations in enzymatic assays and cell monolayer cultures (Graf et al., 2008a; Schnute et al., 2012). As our model is an organoid, we used higher concentrations of inhibitor (100 nM and 1 μM for CERK, and 250 nM and 1 μM for SPHK1), well within the range of specificity. The final concentration of DMSO in the culture medium was 0.01%, which had no detectable effect on otic vesicle cultures.

### Reverse-Transcriptase Quantitative PCR of Gene Expression Patterns

Expression analysis of genes of interest was evaluated in embryonic otic vesicles stages: HH17 (*n* = 22), HH18 (*n* = 15), HH19 (*n* = 10), HH20 (*n* = 9), and HH21 (*n* = 6). Otic vesicles were dissected in cold RNase-free PBS and pooled in 350 μl of RLT buffer (supplied with the RNeasy Plus Mini Kit; Qiagen, Valencia, CA, United States) with 1% b-mercaptoethanol and immediately frozen at −20°C. Total RNA was extracted at the genomic core facility of the IIB (with UNE-EN ISO 9001:2000 certification). The quantity and quality of the RNA was assessed on an Agilent 2100 Bioanalyzer platform (Agilent Technologies, Santa Clara, CA, United States). As per MIQE guidelines (Taylor et al., 2010), only RNA of high purity and integrity was used. cDNA was synthesized using the High Capacity cDNA Reverse Transcription Kit (Applied Biosystems, CA, United States). PCR was performed with an ABI Prism 7900 HT FAST real-time PCR system (ThermoFisher Scientific, Waltham, MA, United States). Specific primers for the chicken genes of ceramide catabolism enzymes and *IGF1* were designed with Primer Express v3.0 software (Thermo Fisher Scientific) and synthesized by Sigma-Aldrich (Supplementary Table 1). Reference genes were selected for normalization considering their stable expression during development (*EMG1*) or under culture conditions (*RPL13*). Results were analyzed by the 2^−ΔΔ^Ct method (Livak and Schmittgen, 2001). Unless otherwise specified, each result represents the mean ± SEM of three independent biological replicates (each with three technical replicates).

### Immunofluorescence, Programmed Cell Death and Proliferation

Otic vesicles were fixed in 4% (w/v) paraformaldehyde for immunostaining and TUNEL labeling. Prior to incubation with the primary antibodies, samples were permeabilized with 1% Triton X-100 (w/v) for 1 h. Samples were then incubated for 1 h in PBS, 0.3% Triton X-100, 1% BSA and 5% normal goat or donkey serum to block non-specific binding sites (blocking solution). Otic vesicles were then incubated overnight at 4°C with primary antibodies (Supplementary Table 2) diluted in blocking solution/PBS (1:1). Bound antibodies were detected by incubating samples for 2 h with species-specific Alexa Fluor-conjugated secondary antibodies (Molecular Probes, Eugene, OR, United States) diluted 1:500 in blocking solution/PBS (1:1). Samples were mounted in Vectashield AntiFade mounting medium with DAPI (Vector Laboratories, Peterborough, United Kingdom).

Apoptosis was studied using the Dead-End^TM^ Fluorometric TUNEL System (Promega, Madison, WI, United States) adapted to whole organ labeling (Frago et al., 2003). The Click-iT^®^ Plus EdU Assay (Invitrogen/Molecular Probes, Eugene, OR, United States) was used to study cell proliferation. EdU (5-ethynyl-2′-deoxyuridine, Invitrogen) was diluted to 100 μM in PBS-1% DMSO and was added to cultures during the last hour before harvest. Finally, otic vesicles were mounted in Vectashield AntiFade mounting medium with DAPI (Vector Laboratories).

### Western Blotting

HH18 otic vesicles (*n* = 15) were cultured overnight in SFM, pre-treated with the CKi for 2 h and then stimulated with IGF-1. To study AKT activation, otic vesicles were incubated with IGF-1 for 30 min, whereas for proliferative cell nuclear antigen (PCNA) analysis, otic vesicles were incubated for 20 h. After the stimulation period, samples were homogenized in ice cold Laemmli buffer containing 50 mM dithiotreitol and protease and phosphatase inhibitor cocktails. The homogenized samples were heated at 95°C for 5 min and frozen immediately. Gels were loaded with solutions containing equal amounts of otic vesicles and resolved by SDS-PAGE on 10 or 12% polyacrylamide gels. Proteins were transferred to polyvinylidene fluoride membranes, which were then blocked using 5% non-fat dried milk or 3% BSA in 10 mM Tris–Hcl, 150 mM NaCl, pH 7.4 and processed for western blotting with the appropriate specific primary antibody (Supplementary Table 2). Secondary antibodies conjugated to horseradish peroxidase were obtained from BioRad (Bio-Rad, Watford, United Kingdom). Antibody binding was revealed by chemiluminescence using the Clarity^TM^ Western ECL substrate and visualized in a ChemiDoc^TM^ XRS + system (both from BioRad). Band intensity of the target proteins was quantified by densitometry with the open source image processing package Fiji (Schindelin et al., 2012). At least three independent experiments were performed per condition. The results are presented as the ratio of phosphorylated protein to total protein.

### Imaging Processing

Images were acquired with a confocal microscope (LSM 710; Zeiss, Jena, Germany) saved as TIFF files and processed using the open-source image processing package Fiji (Schindelin et al., 2012). Cell number and intensity were quantified in compiled confocal microscopy projections and normalized to the control condition, which was given an arbitrary value of 1. The Fiji plugin Simple Neurite Tracer were used to trace and measure neurite length.

### Statistical Analysis

Data are shown as mean ± SEM. Statistical significance was determined with one-way ANOVA followed by the Bonferroni (equal variance) or the Dunnett T3 (unequal variance) test for *post hoc* comparisons. Statistical analysis was performed using SPSS Statistics 26 (IBM SPSS software, version 26.0, Chicago, IL, United States). A *p*-value < 0.05 was considered statistically significant.

## Results

### Genes Encoding the Main Ceramide Metabolism Enzymes and for IGF-1 Are Expressed in the Developing Chicken Inner Ear

**FIGURE 1:**
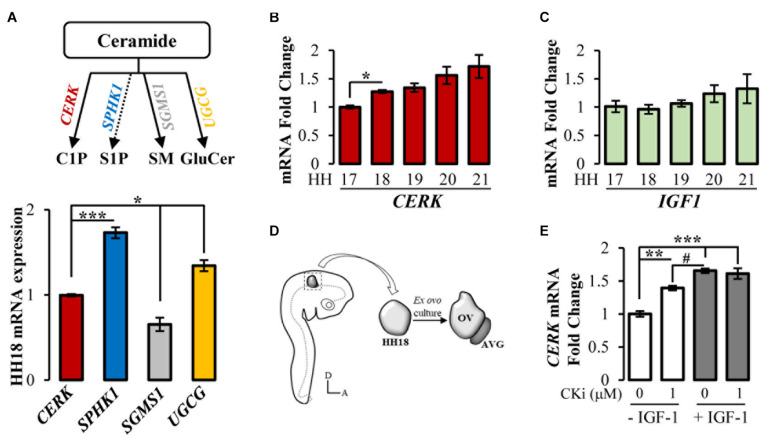
Gene expression of enzymes involved in ceramide processing and *IGF1* expression in early inner ear development in chicken. **(A)** Scheme of the main ceramide metabolism enzymes (upper part) and their gene expression at HH18 related to *CERK*. Data are represented as the mean ± SEM of technical triplicates of five independent experiments. **(B)** Expression of *CERK* and **(C)** *IGF1* from otic vesicle pools obtained from HH17 to HH21 chicken embryos normalized to HH17. Data are represented as mean ± SEM of three independent experiments performed in technical triplicates. **(D**) Schematic cartoon showing the aspect of the *ex vivo* organotypic culture model of otic vesicles dissected from HH18 embryos. The culture mimics *in vivo* otic vesicle (OV) morphogenesis and the development of the acoustic-vestibular ganglion (AVG) in the ventromedial region. **(A)**, anterior; **(D)**, dorsal. **(E)** *CERK* otic vesicle expression levels after 4 h *ex ovo* culture in serum-free medium, 1 μM CKi, 10 nM IGF-1, or IGF-1 plus CKi. Data are represented as mean ± SEM of a technical triplicate from otic vesicle pools (*n* = 15). Statistical significance was calculated by one-way ANOVA followed by Dunnett’s T3 **(A,B)** or Bonferroni’s **(E)** *post hoc* test. **(A)** *p < 0.05, ***p < 0.001 vs *CERK*; **(B)** *p < 0.05 vs HH17; **(E)** **p < 0.01, ***p < 0.001 vs control and ^#^*p* < 0.05 vs IGF-1.

We first studied the expression of genes encoding enzymes involved in ceramide processing (upper scheme in Figure 1A) in chicken otocysts isolated from embryos of stage HH18, the starting stage for organotypic cultures. Taking the expression levels of *CERK* as a reference, data showed that *SPHK1* and *UGCG* (glucosylceramide synthase) transcripts were higher (1.7 ± 0.06 and 1.3 ± 0.06, respectively), whereas *SGMS1* (sphingomyelin synthase 1) was lower (0.65 ± 0.08) (Figure 1A). Extending the study period to HH21, we observed an increase in *CERK* expression from HH17 to HH21, which was significant for HH18 (Figure 1B). By contrast, the levels of the other enzymes remained stable over this period (Data not shown). *IGF1* was also expressed in the otocyst with no significant changes from HH17 to HH21 (Figure 1C).

After confirming the endogenous expression of *CERK and IGF1*, we next studied their interplay by treating *ex vivo* cultures of otic vesicles with 1 μM CKi, 10 nM IGF-1, or their combination, for 4 h (scheme in Figure 1D). Of note, we found that CKi treatment significantly increased *CERK* expression (Figure 1E). Similarly, *CERK* expression was significantly increased by IGF-1 administration, but the combination of IGF-1 plus CKi did not have an additive effect (Figure 1E).

### Inhibition of Ceramide Kinase Obstructs IGF-1-Mediated Survival of Otic Progenitors by Diminishing AKT Activation

**FIGURE 2:**
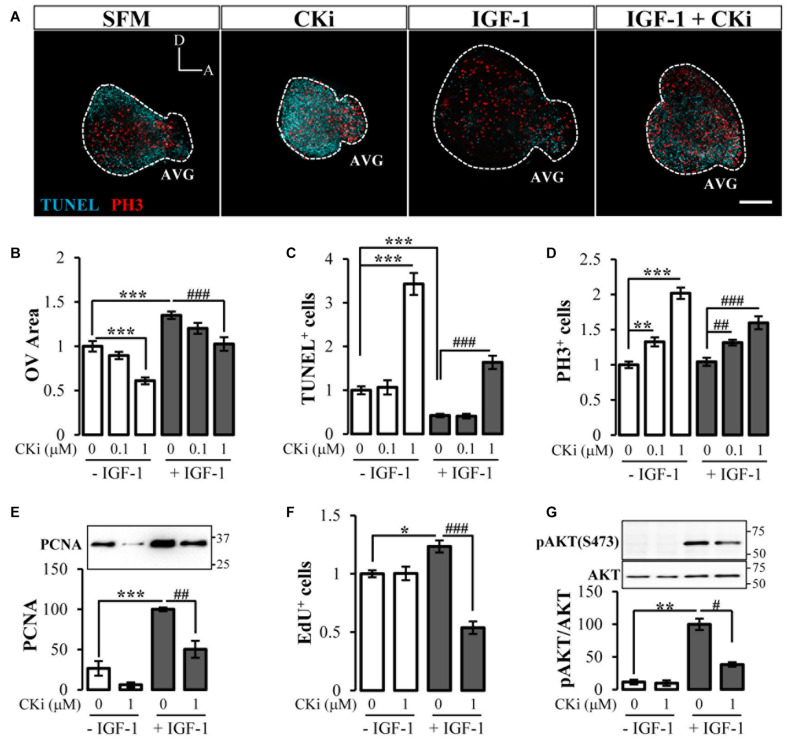
The ceramide rheostat supports *ex ovo* inner ear morphogenesis. **(A)** Otic vesicles were dissected from HH18 embryos and cultured for 20 h in serum-free medium (SFM), 1 μM CKi, 10 nM IGF-1 alone or with CKi. Morphological otic vesicle aspect and immunofluorescence staining for TUNEL (cyan) and PH3 (red) of at least 4 otic vesicle per condition from three independent experiments are shown. Scale bar: 150 μm. Quantification of **(B)** otic vesicle areas, **(C)** TUNEL-positive and **(D)** PH3-labeled cells with CKi (0.1 and 1 μM) with or without IGF-1. Results are shown as the mean ± SEM relative to the SFM condition. **(E)** Effect of CKi (1 μM) on PCNA protein levels from otic vesicles cultured and processed for western blotting. The results are presented as the mean ± SEM of three independent experiments with *n* = 1 otic vesicle per condition relative to the IGF-1 data. A representative blot is shown above. **(F)** Quantification of EdU-positive cells obtained from confocal images and normalized to data from SFM (the first bar). Data are represented as mean ± SEM of *n* = 7 otic vesicles per condition. **(G)** Effect of CKi on the activation of AKT (Ser-473, pAKT) determined by western blotting. The results are presented as the mean ± SEM of four independent experiments with *n* = 15 otic vesicles per condition relative to the IGF-1 data. Representative blots are shown above. Statistical significance was calculated by one-way ANOVA followed by Dunnett’s T3 *post hoc* test **(B–D,G)** or Bonferroni’s *post hoc* test **(E,F)**. *p < 0.05, **p < 0.01, ***p < 0.001 *vs* control; ^#^*p* < 0.05, ^##^*p* < 0.01, ^###^*p* < 0.001 vs IGF-1.

We next investigated the role of CERK in inner ear morphogenesis. Otic vesicles cultured in medium without supplements (SFM) showed early signs of morphogenesis, among others AVG formation, likely due to the presence of endogenous growth factors including IGF-1 (Figure 2A). Otic vesicles cultured with CKi showed a similar shape but were smaller in size (Figure 2A). By contrast the addition of exogenous IGF-1 to cultured otic vesicles increased their size, mimicking the *in vivo* developmental pattern (Figure 2A), and this was partially impaired by co-treatment with CKi (Figure 2A). Figure 2B shows the quantification of otic vesicle area and its dose-dependent response to CKi in the presence or absence of IGF-1. Decrease in size is accompanied by apoptosis (TUNEL-positive cyan dots in Figure 2A). Treatment of otic vesicles with 1 mM CKi significantly increased the number of TUNEL-positive cells (>three-fold with respect to SFM alone), whereas treatment with IGF-1 significantly decreased the number of TUNEL-positive cells (<50%) (Figure 2A; quantification in Figure 2C). Co-treatment of otic vesicles with IGF-1 and CKi increased the number of TUNEL-positive cells with respect to IGF-1 alone (Figures 2A,C), suggesting impairment of the pro-survival effects of IGF-1.

Despite the reduction in size and increase in apoptosis, otic vesicles treated with CKi showed a significant increase in the relative number of mitotic PH3-positive cells/area (Figure 2A; quantification in Figure 2D). IGF-1 treatment expanded the area of mitotic cells but did not increase the ratio of mitotic cells/area (Figures 2A,D). Co-treatment of otic vesicles with IGF-1 and CKi increased the number of mitotic cells above that seen for IGF-1 alone (Figures 2A,D). This CKi-induced increase in the mitotic cells was paradoxical and could be understood as an unwanted cell cycle arrest that might trigger apoptosis, as reported in other cellular contexts (Pastukhov et al., 2014). Thus, we next examined the G1/S-phase associated protein PCNA. Western blotting showed that PCNA levels in otic vesicles cultured in SFM with or without CKi were at least ∼4-fold lower than in vesicles treated with IGF-1, whereas co-treatment with CKi and IGF-1 reduced the PCNA levels by 50% relative to IGF-1 alone (Figure 2E). Finally, we measured DNA synthesis and the cell cycle S-phase by EdU incorporation. As expected, the number of EdU-positive cells was significantly higher in IGF-1-treated vesicles than in SFM cultured vesicles, and this increase was significantly reduced in IGF-1 and CKi co-treated cells (Figure 2F). Overall, these data support the notion that CERK inhibition causes mitotic arrest and induces apoptosis, and that both the proliferative and survival actions of IGF-1 on otic vesicles require CERK activity.

The canonical mediator of cell survival triggered by IGF-1 is the PI3K target AKT (Aburto et al., 2012). We tested the effect of CERK inhibition on IGF-1/AKT signaling by evaluating the phosphorylation of AKT at a conserved serine residue (Figure 2G). AKT phosphorylation (activation) was barely detectable in otic vesicles cultured in SFM treated or not with CKi, as shown by western blotting, whereas IGF-1 treatment markedly increased AKT phosphorylation (∼nine-fold). Vesicles co-treated with IGF-1 and CKi showed decreased levels of AKT activation. CKi failed to trigger the activation of other kinases reported to be downstream of IGF-1 including ERK1/2 (Magariños et al., 2010) or kinases targeted by ceramide, such as the stress-activated protein kinases (SAPK/JNK) and p38α (Morad and Cabot, 2013) (Data not shown).

### CERK Inhibition Compromises AVG Development

**FIGURE 3:**
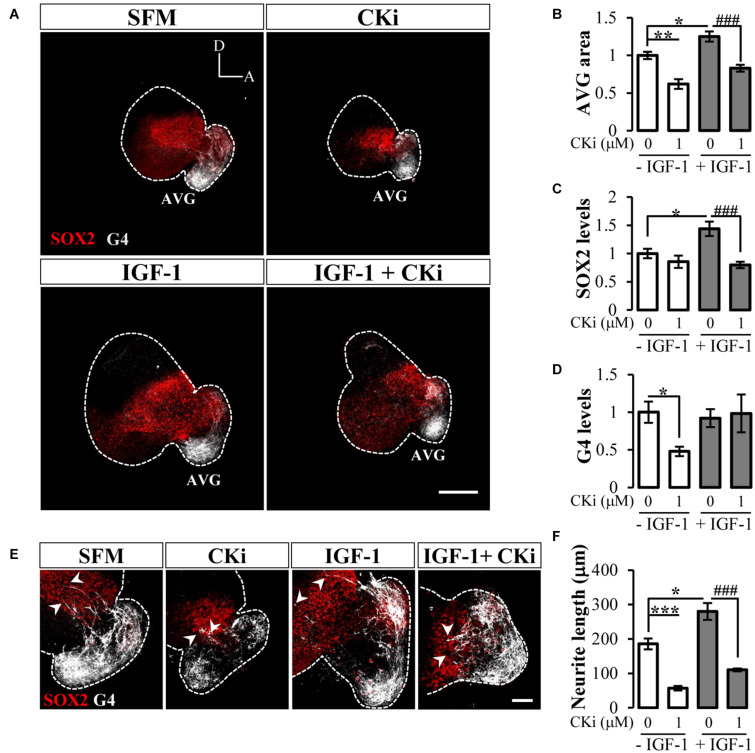
CERK inhibition disrupts early otic neurogenesis. **(A)** Confocal images of representatives otic vesicles immunostained with SOX2 (red) or G4 (white) after 20 h of culture in serum-free medium (SFM), 1 μM CKi, 10 nM IGF-1, or IGF-1 plus CKi. Scale bar: 150 μm. Histograms represent the quantification of the AVG size **(B)**, SOX2 levels in otic vesicle **(C)** and G4 levels in AVG **(D)** normalized to data for SFM. Data are represented as mean ± SEM of two independent experiments, *n* = 8 otic vesicles per condition. **(E)** Magnification showing the AVG area with arrowheads pointing to neurite processes (white). Scale bar: 50 μm. **(F)** Histogram represents neurite length mean ± SEM from *n* = 8 otic vesicles per condition. Statistical significance was calculated by one-way ANOVA followed by Bonferroni’s *post hoc* test **(B–D)** or Dunnett’s T3 *post hoc* test **(F)**. *p < 0.05, **p < 0.01, ***p < 0.001 vs control; *^###^p* < 0.001 vs IGF-1.

Our former results showing that inhibition of CERK activity impacts otic vesicle morphogenesis prompted us to explore its particular effect on AVG development. Transition from otic neuroblasts to neurons occurs through sequential stages that can be followed by specific population biomarkers as the SOX2-sensory progenitor marker and G4-neuronal AVG population marker (Camarero et al., 2003). Otic vesicles treated with CKi had a significant reduced AVG *vs.* SFM (Figure 3A; quantification in Figure 3B). By contrast, IGF-1 administration increased the size of the AVG, which was counteracted by the addition of CKi (Figure 3A; quantification in Figure 3B). IGF-1 treatment also significantly increased the number of SOX2-positive sensory progenitors, whereas co-treatment with CKi significantly reduced the IGF-1-induced increase in SOX2 staining (Figure 3A; quantification in Figure 3C). The addition of CKi in absence of IGF-1 did not produce any significant change in SOX2 staining with respect to SFM (Figure 3C). In addition, CKi treatment significantly reduced G4 staining (Figure 3D).

To further investigate AVG differentiation, we measured the length of neurites exiting the AVG to reach the sensory otic epithelium (Figures 3E,F). Otic vesicles cultured under basal (SFM) conditions or stimulated with IGF-1 had elongated fibers extending to the sensory epithelium (186 ± 16 μm and 280 ± 24 μm, respectively) (arrowheads in Figure 3E). These fibers were shorter in length in the presence of CKi (57 ± 6 μm) which significantly diminish the IGF-1-induced effect on AVG neuritogenesis (110 ± 4 μm) (Figure 3F).

### The Inhibition of SPHK1 by PF-543 Has no Effect on Early Inner Ear Development

**FIGURE 4:**
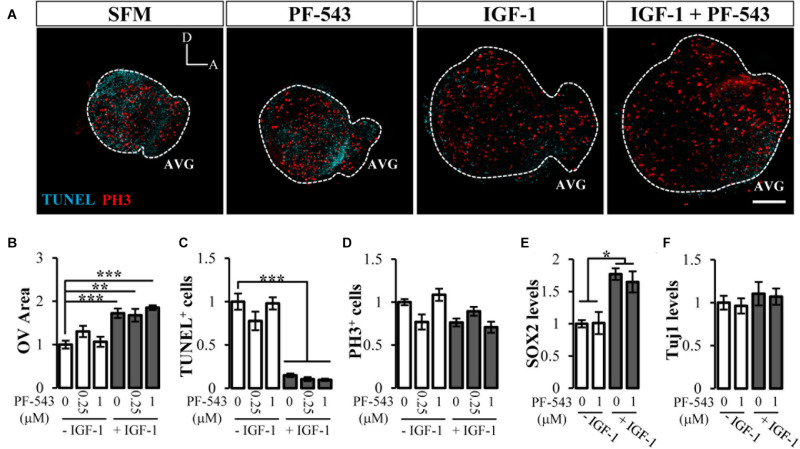
Inhibition of endogenous SPHK1 has no effect on early inner ear development. **(A)** Morphological aspect of organotypic cultures after 20 h in serum-free medium (SFM), 1 μM PF-543, 10 nM IGF-1, IGF-1 plus PF-543. Immunostaining with TUNEL (cyan) and PH3 (red) of at least 5 otic vesicle per condition. Scale bar: 150 μm. **(B)** Quantification of otic vesicle area, **(C)** TUNEL-positive or **(D)** PH3-labeled cells in the presence or absence of PF-543 (0.25 and 1 μM) and IGF-1 (10 nM). **(E)** SOX2 intensity **(F)** and Tuj-1 intensity as neurogenesis markers normalized to data for SFM. Data are represented as mean ± SEM from at least *n* = 4 otic vesicles per condition. Statistical significance was calculated by one-way ANOVA followed by Bonferroni’s **(B,E)** or Dunnett’s T3 *post hoc* test **(C)**. *p < 0.05, **p < 0.01, ***p < 0.001 vs control.

To address whether the observed actions of CERK were specific to ceramide or were due to ceramide interconversion with other bioactive sphingolipids, we investigated the potential role of S1P in inner ear morphogenesis because of its reported developmental actions (Romero-Guevara et al., 2015). Like the study of CERK, we used a specific inhibitor of SPHK1 activity, PF-543 (Schnute et al., 2012). Otic vesicles were cultured for 20 h with 250 nM or 1 μM PF-543 in the absence or presence of IGF-1. The addition of PF-543 failed to alter morphogenesis or otic vesicle area under any of the conditions studied (Figure 4A; quantification in Figure 4B). No significant differences were found for apoptosis or proliferation measured by TUNEL and PH3 staining, respectively (Figure 4A; quantification in Figures 4C,D). SPHK1 inhibitor had no impact on AVG neurogenesis since no differences were observed neither in SOX2 levels (Figure 4E) nor in the neuronal maturation marker β-III-tubulin (Tuj1) (Figure 4F). These results thus suggest that the inhibition of SPHK1 activity has no impact on the actions of IGF-1 on early otic development.

## Discussion

Hearing loss is a disabling disease whose prevalence is increasing worldwide due to an aging population World Health Organization (2020). Deafness and Hearing Loss. World Health Organization Fact Sheets. https://www.who.int/news-room/fact-sheets/detail/deafness-and-hearing-loss). Novel therapeutic approaches are focused on the identification of survival molecules important for normal inner ear development. In this respect, sphingolipids are promising candidates because of their abundance in neural membranes and their role as regulators of brain homeostasis (Mencarelli and Martinez–Martinez, 2013; Olsen and Færgeman, 2017). In fact, alterations in sphingolipid metabolism are associated with age-related hearing loss and neurological diseases (Romero-Guevara et al., 2015; Pujol-Lereis, 2019). Here we show, for the first time, the expression of the principal enzymes involved in ceramide metabolism in the developing chicken inner ear which suggests the presence of endogenous sphingolipids. To date, CERK is the only enzyme known to produce C1P and is therefore key for controlling cellular ceramide levels (Hannun and Obeid, 2018). The lower levels of *CERK* at HH17 with respect to HH18 could be related to an increase in endogenous ceramide that promotes cell death, a mechanism necessary to detach the otocyst from the surface ectoderm, a developmental process that takes place in this stage (León et al., 2004).

We also show that CERK inhibition affects inner ear development. Previously we described the presence of ceramide in otic vesicles (Frago et al., 1998). In the present study, we used a specific inhibitor of CERK, NVP-231 (called CKi throughout the text to simplify) that blocks the conversion of endogenous ceramide to C1P (Graf et al., 2008a). CKi increased cell cycle arrest and apoptosis in the otocyst. A similar effect of this inhibitor has been described in cancer cells (Pastukhov et al., 2014). The specificity of the inhibitor in preventing the phosphorylation of ceramides has been demonstrated by comparison with other compounds of similar chemical structure but modified to abolish their inhibitory potential (Graf et al., 2008a, 2009; Pastukhov et al., 2014). Our results with CKi suggest that the transformation of endogenously produced ceramide to C1P is necessary for proper inner ear morphogenesis.

We reported earlier the expression of IGF-1 in HH19 chicken embryos by *in situ* hybridization (Camarero et al., 2003). Here we show that *IGF-1* is expressed from HH17 to HH21, and that expression levels are maintained. Because the addition of IGF-1 to otic cultures increased the expression of *CERK*, we hypothesized that the cytoprotective role of IGF-1 could be due to the activation of CERK, which is consistent with the loss of the pro-survival actions of IGF-1 by CKi. This is supported by the activation of AKT by IGF-1 and its inhibition by CKi, holding up the role of C1P in otic cell survival opposed to the pro-apoptotic role of ceramide. The link between sphingolipids and the IGF-1/AKT pathway in the modulation of cell survival, aging and age-related neurodegenerative disorders has been recently reported (Jȩśko et al., 2019). Indeed, C1P protects against cisplatin-induced ototoxicity in mouse cochlear explants through the activation of AKT and MAPK pathways (Le et al., 2016). Our results thus suggest that the effects of IGF-1 on CERK and AKT might determine the survival of epithelial otic progenitors.

CERK inhibition also alters AVG neurogenesis. As previously reported, the addition of exogenous IGF-1 yields a larger number of SOX2-positive epithelial precursors in the otic vesicle (Aburto et al., 2012). Our data show that CERK inhibition not only reduces the neural pool promoted by exogenous IGF-1, but also impairs the outgrowth of processes from the remaining neurons. The inhibition of CERK has been recently related to Müller glial migration (Vera et al., 2021). Our findings also point to the involvement of CERK activation in AVG formation and neurite outgrowth.

The expression of *SPHK1* in otic progenitors suggests a role in inner ear development. In fact, the expression level of *SPHK1*, the only isoform able to produce S1P in chicken, is higher than that of CERK. S1P maintains murine auditory neuroprogenitors (Bruno et al., 2017) and an S1P transporter was shown to be involved in childhood deafness (Ingham et al., 2019). However, embryos of *SPHK1/SPHK2* double mutant knockout mice have normal inner ear development (Meng et al., 2011). The results presented here on SPHK1 inhibition do not support a key role for S1P in inner ear development as we had previously described using a synthetic S1P analog in *ex vivo* otic vesicle cultures (Frago et al., 1998).

In summary, here we show that inhibition of CERK activity impairs the development of the chicken inner ear otocyst. Our data would suggest a crucial role for CERK activation in the IGF-1 prosurvival actions on otic vesicle epithelial cells and in the establishment of the spatiotemporal control of neurogenesis. This conclusion comes from the following evidence: (i) the expression of the main enzymes related to ceramide homeostasis in the developing inner ear; (ii) the upregulation of *CERK* by IGF-1 in *ex vivo* organotypic cultures; (iii) the CKi-mediated impairment of IGF-1 prosurvival actions through inactivation of AKT; and (iv) the defects in AVG neurogenesis by CERK inhibition.

## Data Availability Statement

The datasets presented in this study can be found in online repositories. The names of the repository/repositories and accession number(s) can be found below: The data for this study will be deposited in DIGITAL.CSIC (https://digital.csic.es), the institutional repository of the Spanish National Research Council.

## Ethics Statement

Ethical review and approval was not required for the animal study because all procedures were performed with chicken embryos of less than 4 days of development and DIRECTIVE 2010/63/EU does not consider fetal forms before last third of their development.

## Author Contributions

All authors have made a significant contribution to the work, conceiving, and designing the experiments. YL was the main responsible for the research, analysis, writing, and figure layouts. MM and IV-N contributed to research and figure layouts. IV-N and MM were responsible for resource acquisition. All authors revised the manuscript and figures, provided comments, and approved the final version.

## Conflict of Interest

The authors declare that the research was conducted in the absence of any commercial or financial relationships that could be construed as a potential conflict of interest.

## Abbreviations

AVG: acoustic-vestibular ganglion
C1P: ceramide-1-phosphate
CERK: ceramide kinase
CKi: ceramide kinase inhibitor NVP-231
HH: Hamburger and Hamilton
IGF-1: insulin-like growth factor 1
S1P: sphingosine-1-phosphate
SFM: serum-free medium.
