# Supplementary material for: Ceramide Kinase Inhibition Blocks IGF-1-Mediated Survival of Otic Neurosensory Progenitors by Impairing AKT Phosphorylation
Source: Front Cell Dev Biol. 2021 Jun 4;9:678760. doi: 10.3389/fcell.2021.678760 (PMC8220815; doi:10.3389/fcell.2021.678760)
Supplement: Supplementary file 1 [file Table_1.DOCX]

Supplementary Material

**Supplementary Table 1.** Chicken primer sequences used for RT-qPCR

| Gene | GenBank Accession Nº | Description | Primer sequences 5’-3’ (forward/reverse) |
| --- | --- | --- | --- |
| *CERK* | NM_001031340.1 | Ceramide kinase | CACTCTTCAGCTTGGCTTCTATTTC  AAGTTGTCCTTAGCATGGTTAGCA |
| *EMG1* | NM_001278069.1 | Essential for Mitotic Growth 1 | GACATCACCCACCAGAGTCTTCT  AGGCCAGCCCGATTCAG |
| *IGF1* | NM_001004384.2 | Insulin like growth factor 1 | CCAGCAGTAGACGCTTACACC  CTCCTCAGGTCACAACTCTGG |
| *RPL13* | NM_204999.1 | 60S ribosomal protein L13 | AACTCAAGATGGCAACTCAGC  AAGGCCTTGAAGTTCTTCTCC |
| *SGMS1* | NM_204390.2 | Sphingomyelin synthase 1 | CACCGTCATATTAACTCTCACCTACTTATT  TCCAGCAAAGCCAGTGATACC |
| *SPHK1* | XM_015295300.1 | Sphingosine kinase 1 | TGCTGGCTGATGCAGACATC TCTTCATCCCGCACCTTCTC |
| *UGCG* | XM_424914.6 | Glucosylceramide synthase | TCTTCGGTCTGATCCTCTTTGTC  TGGAGGCGTGTGTAGATGATG |

Chicken gene data are from <http://www.ensembl.org/Gallus_gallus>.

**Supplementary Table 2.** List of primary antibodies and working conditions.

| Antibody (Anti-) | Host | Supplier/ Cat. # | Working Dilution | Use |
| --- | --- | --- | --- | --- |
| AKT | m | Santa Cruz/ sc-5298 | 1:1000 | WB |
| G4 glycoprotein | rb | Dr. E. de la Rosa | 1:500 | IF |
| p38 MAPK | rb | Cell Signaling/ 9212 | 1:1000 | WB |
| p44/42 MAPK | rb | Cell Signaling/9102 | 1:1000 | WB |
| PCNA | m | SIGNET/523-01 | 1:1000 | WB |
| Phospho-AKT (S473) | rb | Cell Signaling/9271 | 1:1000 | WB |
| Phospho-Histone-H3 (PH3) | rb | Upstate/06-570 | 1:200 | IF |
| Phospho-JNK (T183/Y185) | rb | Promega/ V-793 | 1:1000 | WB |
| Phospho-SAPK/JNK (T183/Y185) | m | Cell Signaling/9255 | 1:1000 | WB |
| Phospho-p38 MAPK (T180/Y182) | m | Cell Signaling/9216 | 1:1000 | WB |
| Phospho-p44/42 MAPK (T202/Y204) | m | Cell Signaling/ 9106 | 1:1000 | WB |
| SAPK/JNK | rb | Cell Signaling/ 9252 | 1:1000 | WB |
| SOX2 | g | Santa Cruz/ sc-17320 | 1:300 | IF |
| Tuj-1 (β-III tubulin) | rb | Covance/ PRB-435P | 1:1000 | IF |

Host: g, goat; m, mouse; rb, rabbit. Technique: IF, immunofluorescence; WB, western blotting.
